# Prognostic Factors for Distress After Genetic Testing for Hereditary Cancer

**DOI:** 10.1007/s10897-015-9894-9

**Published:** 2015-10-16

**Authors:** Jan S. Voorwinden, Jan P. C. Jaspers

**Affiliations:** Department of Medical Psychology, University of Groningen, University Medical Center Groningen, PO Box 30.001, 9700 RB Groningen, The Netherlands

**Keywords:** Genetic testing, Hereditary cancer, *BRCA*, Lynch syndrome, Distress, Cancer worry, Prognostic factors

## Abstract

The psychological impact of an unfavorable genetic test result for counselees at risk for hereditary cancer seems to be limited: only 10–20 % of counselees have psychological problems after testing positive for a known familial mutation. The objective of this study was to find prognostic factors that can predict which counselees are most likely to develop psychological problems after presymptomatic genetic testing. Counselees with a 50 % risk of *BRCA1/2* or Lynch syndrome completed questionnaires at three time-points: after receiving a written invitation for a genetic counseling intake (T1), 2–3 days after receiving their DNA test result (T2), and 4–6 weeks later (T3). The psychological impact of the genetic test result was examined shortly and 4–6 weeks after learning their test result. Subsequently, the influence of various potentially prognostic factors on psychological impact were examined in the whole group. Data from 165 counselees were analyzed. Counselees with an unfavorable outcome did not have more emotional distress, but showed significantly more cancer worries 4–6 weeks after learning their test result. Prognostic factors for cancer worries after genetic testing were pre-existing cancer worries, being single, a high risk perception of getting cancer, and an unfavorable test result. Emotional distress was best predicted by pre-existing cancer worries and pre-existing emotional distress. The psychological impact of an unfavorable genetic test result appears considerable if it is measured as “worries about cancer.” Genetic counselors should provide additional guidance to counselees with many cancer worries, emotional distress, a high risk perception or a weak social network.

## Introduction

The psychological impact of an unfavorable genetic test result for counselees at risk for hereditary cancer appears to be limited (Beran et al. 2008; Collins et al. 2007; Halbert et al. 2011; Hamilton et al. 2009; Smith et al. 2008). It is assumed that treatment options, such as preventive surgery or regular surveillance to detect cancer at an early stage, reassure most counselees. However, a small group (between 10 and 20 %) experience psychological problems after receiving an unfavorable DNA test result (Coyne et al. 2000; Esplen et al. 2001; Hopwood et al. 1998). Individuals in this group were studied to find prognostic factors that can predict which counselees are most likely to have psychological problems after genetic testing. Genetic counselors could then provide extra guidance and, if necessary, extra psychosocial support to these counselees.

The best-known prognostic factor for psychological problems after genetic testing for hereditary cancer is the presence of pre-existing psychological distress (Gritz et al. 2005; Lodder et al. 2001; Reichelt et al. 2004; Smith et al. 2008; Van Oostrom et al. 2003). However, few studies have examined a wide range of possible prognostic factors. Van Oostrom et al. (2007) found that pre-existing psychological distress, an unfavorable DNA test result, complicated grief, relatives with cancer, and strong emotional illness representations were significant predictors for psychological problems after genetic testing. It is noteworthy that pre-existing psychological distress had more influence than receiving an unfavorable result. There were also prognostic variables with less predictive value: low disease coherence (i.e. experiencing the disease as uncontrollable), a passive or distraction-seeking coping style, and a closed style of communication within the family. Another population in which a wide range of prognostic factors was examined consisted of counselees with a *BRCA* mutation who had chosen regular surveillance as a preventive option (Den Heijer et al. 2013; Gopie et al. 2012). Den Heijer et al. (2013) concluded that pre-existing psychological distress, relatives with cancer, a passive and distraction-seeking coping style, excessive self-examination of breasts, and a high risk perception were predictive of psychological distress in the long term (5–8 years later), whereas reassuring thoughts as a coping style was a protective factor. In a review article by Gopie et al. (2012), it appeared that a young age (under 40 years), high risk perception, pre-existing psychological distress, a passive way of coping, little social support, and family members with cancer were predictive of psychological problems and/or a reduced quality of life.

Several potential prognostic factors and the extent to which they could predict the occurrence of psychological problems after genetic testing were explored. Given earlier reports, it was expected that pre-existing psychological distress, an unfavorable result, and a high risk perception would be prognostic variables (Den Heijer et al. 2013; Gopie et al. 2012; Van Oostrom et al. 2007). Psychological distress was measured using both a general measure, “emotional distress”, and a specific measure, “worries about cancer.” The other variables that have been examined were age, knowledge about the disease, perceived personal control regarding the counseling process, the intention of having preventive surgery if the test result was unfavorable, type of cancer, education level, having children, having daughters, and marital status. Less is known about these variables. In some studies, a young age (Gopie et al. 2012) and perceived personal control (Van Oostrom et al. 2007) were prognostic variables. In other types of hereditary cancer, a lower educational level and being childless gave more distress (Gopie et al. 2012). Although marital status had no influence in one study (Van Oostrom et al. 2007), another found that counselees who were single were more depressed (Gopie et al. 2012). The type of cancer (*BRCA* or Lynch syndrome) had no influence in one study (Van Oostrom et al. 2007).

### Purpose of the Study

This research deals with a wide range of prognostic factors that can predict which counselees are most likely to develop psychological problems after presymptomatic genetic testing for hereditary cancer. Published data on this topic are limited: most studies have measured only a small number of prognostic variables. Prediction of possible psychological impact is important in health care, as it helps to target interventions to those counselees that are likely to benefit most. Another point of interest is that earlier research have shown that the psychological impact of an unfavorable presymptomatic genetic test result is limited. In many of these studies generalized distress has been measured. In this research also cancer-specific distress will be measured, as this outcome measure could better represent the type of distress many counselees experience. This research has an additive value by looking at many prognostic variables and by comparing generalized and specific distress.

Based on the earlier mentioned findings two hypotheses were tested:

1. After genetic testing for hereditary cancer, counselees with an unfavorable DNA test result will *not* have significantly more psychological problems than those with a favorable result.
2. The prognostic factors that are most predictive of psychological problems after genetic testing for hereditary cancer are an unfavorable result, pre-existing worries about cancer, pre-existing emotional distress, and a high risk perception of getting cancer.

## Methods

### Participants

**Fig. 1:**
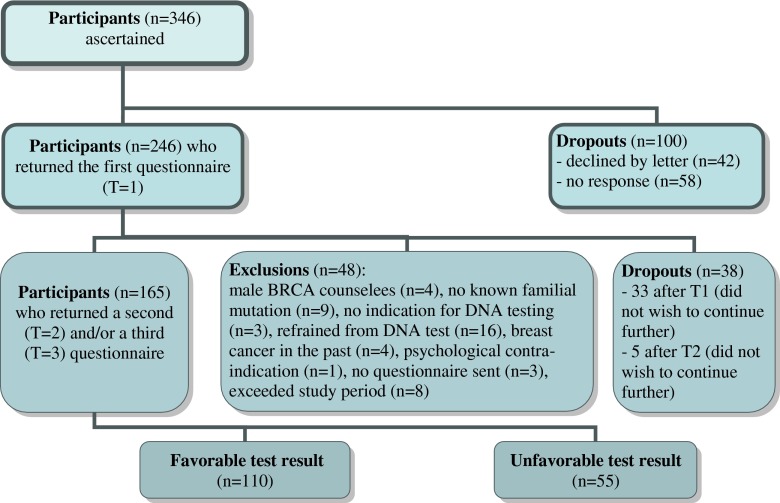
Flow chart of the study design

Around 90 % of the participants had a 50 % chance of having a known familial *BRCA1/2* gene mutation or Lynch syndrome (the rest had a 25 % or 12,5 % chance). All participants were 18 years and older, pre-symptomatic and spoke sufficient Dutch to complete the questionnaires. The Lynch syndrome group consisted of both men and women, while the *BRCA 1/2* group comprised only women. Figure 1 shows a flow chart of the study design. Out of 346 counselees who were invited to participate, 246 returned questionnaire T1 (71.1 %). In total 165 counselees returned questionnaire T2 and/or T3 (47.7 %). 127 counselees returned all three questionnaires. After T1, 48 counselees were excluded at the intake session and 33 drop-outs occurred during the course of the study.

### Instrumentation

#### Socio-Demographic Data and DNA Test Result

Socio-demographic data were gathered in the first questionnaire (T1). In the second (T2) and third (T3) questionnaires, the counselees were asked whether their test result was favorable or unfavorable.

#### Personal Control

The Perceived Personal Control (PPC) questionnaire (Berkenstadt et al. 1999) measures how much personal control people experience as a counselee. The instrument has been validated for a Dutch setting (Smets et al. 2006). All nine items were summed and used as an indicator of personal control. The internal consistency for this study population was good (Cronbach’s α of 0.82 (T1), 0.85 (T2) and 0.85 (T3)).

#### Knowledge About the Disease, Risk Perception, and Decision-Making

These questionnaires were based on the Breast Cancer Risk Communication study (Ockhuysen-Vermey et al. 2008). The knowledge questionnaire has eight statements on hereditary cancer and three questions about risk percentages for ‘cancer in general’, ‘a predisposition to hereditary cancer’ and ‘getting cancer when having a predisposition to hereditary cancer’. The total score could range from 0 to 11. A higher score indicates more knowledge about hereditary cancer. The internal consistency for this study population was okay (Cronbach’s α of 0.65 (T1), 0.60 (T2) and 0.61 (T3)). The risk perception questionnaire has seven items. Participants had to state how risky they found the chance of getting cancer and the anxiety they experienced about it on a 7-point scale. The range of the total score is 0–49. A higher score is indicative of higher risk perception. The internal consistency for this study population was good (Cronbach’s α of 0.85 (T1), 0.86 (T2) and 0.85 (T3)). The decision-making process was studied by asking participants to indicate whether they would be willing “to have a preventive operation” if they should receive an unfavorable test result, with answers possible on a 5-point scale ranging from “definitely not” to “certainly.”

#### Psychological Functioning

Emotional distress was measured with the 12-item version of the General Health Questionnaire (GHQ-12) (Goldberg and Williams 1988). The GHQ-12 is a widely-used, standardized questionnaire. The range of the total score is 0–12. A total score of 2 or higher is an indication of emotional distress. The internal consistency for this study population was good (Cronbach’s α 0.89 (T1), 0.90 (T2) and 0.91 (T3)). Concerns about developing cancer were measured with four items from the Cancer Worry Scale (CWS) (Lerman et al. 1991). The CWS is often used for this type of research despite its limited psychometric qualities (Helmes et al. 2006). The range of the total score is 0–4. A higher score is indicative of greater worries about getting cancer. The internal consistency of the CWS for this study population was low (Cronbach’s α 0.59 (T1), 0.52 (T2) and 0.50 (T3)), which means that these results should be interpreted with caution.

### Procedure

Participants were recruited by genetic counsellors between September 2007 and December 2010. All counselees referred to the clinical genetics department at the University Medical Center Groningen (UMCG) because a germline mutation in the *BRCA1* or *BRCA2* gene or in one of the Lynch syndrome-associated genes had been identified in their family, were eligible. This means that counselees from families with unexplained variants were not included. Together with an invitation letter for the intake session and the usual general information leaflets on the procedure of genetic counseling and on family history retrieval, the counselees were sent information about our study with an informed consent form and a first questionnaire. The rationale for the study was formulated in the invitation letter. This rationale was based on prior research about tailoring the disclosure of DNA test results to the needs and wishes of the counselees (Voorwinden et al. 2012). The data of that research has been used for this study. It was made clear that participation in the study would in no way affect their eligibility for DNA testing and counselling. Participants who agreed to participate knew they had to complete questionnaires at three time points: before the intake (T1), 2–3 days after receiving the DNA result (T2), and 4–6 weeks later (T3). There was a period of 2–6 weeks between the invitation and intake session, and 5–7 weeks between the intake and result of the DNA test. Counselees who had not returned an informed consent form 2 weeks before the intake were reminded by phone that they could take part in the study. The study was approved by the UMCG institutional board.

### Data Analysis

First, the pre-test (T1) scores of the demographic and test variables were compared for counselees with a favorable and an unfavorable result. Second, differences in these variables were checked between the participants and dropouts. Chi-square tests for the demographic data and independent *t*-tests for age and test variables were used. The first hypothesis was tested with a covariance analysis in order to correct for the group differences that were found in the pre-test. For the outcome measure, the total scores of the GHQ and CWS were used separately. For the second hypothesis, all the potentially prognostic factors were entered individually into a univariate regression analysis to determine whether there was a significant relationship with the outcome variables. The potentially prognostic factors with a *p*-value of ≤ 0.05 were included in a multiple regression analysis, for which a backward eliminating procedure was used. Multicollinearity was verified by checking whether the VIF (Variance Inflation Factor) values were below 10 and if the average VIF value was around 1. The predictive value of the final model was determined by the percentage of explained variance (adjusted *R*^*2*^). To determine the extent to which the model was able to predict which individuals would have heightened concerns about cancer and emotional distress, a Receiver Operator Characteristic (ROC) curve was calculated. The “area under the curve” (AUC) of a ROC curve indicates how well the model is able to correctly predict which individuals will develop psychological problems. An AUC of 1.0 would mean that the model can identify perfectly all the individuals with emotional distress or concerns about cancer.

## Results

### Pre-Test Analyses

Of the 165 participants, 110 received a favorable test result and 55 an unfavorable one. This imbalance can be explained by age-related penetrance (which means that older pre-symptomatic counselees have a lower chance of an unfavorable result because they are still unaffected). Table 1 shows the demographics and test variables of the pre-test analyses (T1). Counselees with a favorable result were significantly older than counselees with an unfavorable result [M = 47.00 years, SD = 13.1 vs. M = 40.70 years, SD = 13.5, t(160) = −2.87, *p* < .05] and a significantly greater percentage had children [86 % vs. 67 %, *χ*^2^(1) = 7.41, *p* < .05]. With respect to the test variables, counselees with a favorable result had a significantly lower risk perception [M = 32.30, SD = 7.73 vs. M = 35.71, SD = 5.33, t(136) = 3.18, *p* < .05].

**Table 1:** The demographic and test variables of the participants before they knew their DNA test result (T1)

	Favorable result (*N* = 110)		Unfavorable result (*N* = 55)		
	%	*n*	%	*n*	*p*
Demographic data					
Age^ab^					
Mean (SD)	47.00 (13.1)	108	40.70 (13.5)	54	0.01*
Gender					
Women	95.5	105	89.1	49	0.18
Men	4.5	5	10.9	6	
Marital status					
Married	76.4	84	60.0	33	0.06
Unmarried	13.6	15	18.2	10	
Living together	10.0	11	21.8	12	
Children					
Yes	85.5	94	67.3	37	0.01*
No	14.5	16	32.7	18	
Cancer type					
BRCA1/2	86.4	95	76.4	42	0.13
Lynch syndrome	13.6	15	23.6	13	
Education					
Primary school	1.8	2	1.8	1	0.33
Secondary school	20.9	23	18.2	10	
Low vocational education	14.5	16	3.6	2	
Middle vocational education	37.3	41	47.3	26	
Higher vocational education	16.4	18	21.8	12	
University education	9.1	10	7.3	4	
	mean (SD)	*n*	mean (SD)	*n*	*p*
Test variables					
PPC^*a*^	1.37 (0.36)	104	1.49 (0.39)	54	0.06
Knowledge^*a*^	6.06 (2.41)	99	6.27 (2.23)	52	0.61
Risk perception^*a*^	32.30 (7.73)	101	35.71 (5.33)	51	0.01*
Decision making^*a*^	3.42 (1.17)	109	3.69 (1.16)	54	0.18
CWS^*a*^	0.30 (0.66)	109	0.46 (0.75)	54	0.16
GHQ^*a*^	1.42 (2.57)	108	1.18 (2.13)	55	0.56

PPC Perceived Personal Control; CWS Cancer Worry Scale; GHQ General Health Questionnaire

^a^These variables have missing values

^b^Mean and SD are shown instead of a percentage

* = *p* ≤ 0.05

### Dropouts

The 160 participants who had returned two or more questionnaires were compared with the 38 dropouts regarding demographics and test variables seen at T1 (the five dropouts after T2 were not considered as participants for this analysis). There were no significant differences. The DNA test results of the 33 participants who dropped out after T1 are unknown, because the result was only asked in T2. Of the five dropouts after T2, four had an unfavorable test result and one had a favorable result.

### Research Hypotheses

- *Hypothesis 1. After genetic testing for hereditary cancer, counselees with an unfavorable DNA test result will not have significantly more psychological problems than those with a favorable result.*

Emotional distress did not differ between counselees with a favorable and unfavorable result immediately after they learned the result (T2) or 4–6 weeks later (T3). However, regarding participants’ worries about cancer, the genetic test result did have an influence, but only after 4–6 weeks (T3). Counselees with an unfavorable outcome were more concerned about cancer *F*(1, 123) = 7.19, *p* < .05, *r* = .23 (see Table 2). Thus, hypothesis 1 was partially supported.

**Table 2:** Results of hypothesis 1

Outcome measure	Genetic Test Result	Mean	Std. Deviation	N	Sig. Level
CWS T2	Unfavorable	0.83	0.99	47	0.08
	Favorable	0.42	0.76	91	
CWS T3	Unfavorable	0.63	0.83	41	0.01*
	Favorable	0.25	0.60	87	
GHQ T2	Unfavorable	2.30	2.86	46	0.64
	Favorable	1.84	3.08	91	
GHQ T3	Unfavorable	1.74	2.61	42	0.10
	Favorable	1.00	2.65	87	

* = *p ≤ 0.05*

- *Hypothesis 2. The prognostic factors that are most predictive of psychological problems after genetic testing for hereditary cancer are an unfavorable result, pre-existing worries about cancer, pre-existing emotional distress, and a high risk perception of getting cancer.*

First, a univariate regression analysis was performed for each potentially prognostic variable to see if there was a significant relationship with any of the outcome variables (see Table 3). This analysis showed which variables had the most influence on cancer worries and emotional distress after genetic testing. Only the significant values are shown (*p* ≤ .05). The patient characteristics with a significant relationship were included in a multiple regression analysis. With the use of a backward eliminating procedure, we explored which prognostic factors were the best predictors of cancer worries and emotional distress after genetic testing (see Table 4).

**Table 3:** Univariate regression analyses on the influence of patient characteristics on psychological problems after genetic testing

	β	*p*-value	R^2^	β	*p*-value	R^2^
Outcome measure	CWS T2			CWS T3		
Cancer worries						
Significant prognostic variables						
DNA result	−0.20	0.01	0.04	−0.25	0.00	0.05
Pre-existing cancer worries	0.50	0.00	0.24	0.36	0.00	0.12
Pre-existing emotional distress	0.34	0.00	0.11	0.19	0.03	0.03
Risk perception	0.37	0.00	0.13	0.20c	0.02	0.03
Age	−0.23	0.00	0.05			
Decision making	0.17	0.04	0.02			
Outcome measure	GHQ T2			GHQ T3		
Emotional distress						
Significant prognostic variables						
DNA result				−0.17	0.05	0.02
Pre-existing cancer worries	0.40	0.00	0.15	0.29	0.00	0.08
Pre-existing emotional distress	0.47	0.00	0.22	0.26	0.00	0.06
Risk perception	0.17	0.04	0.02			
Decision making				0.17	0.04	0.02
Being single	0.18	0.03	0.03			

β standardized regression coefficient; R^2^ was adjusted for shrinkage

**Table 4:** Significant prognostic factors on psychological problems after genetic testing determined by multiple regression analysis

	β	*p*-value	R^2^	AUC
Cancer worries				
Outcome measure CWS T2				
Pre-existing cancer worries	0.45	0.00	0.31	0.80
Risk perception	0.26	0.00		
Being single	−0.19	0.01		
Outcome measure CWS T3				
Pre-existing cancer worries	0.44	0.00	0.23	0.77
DNA result	−0.24	0.00		
Being single	−0.14	0.10		
Emotional distress				
Outcome measure GHQ T2				
Pre-existing emotional distress	0.43	0.00	0.18	0.72
Outcome measure GHQ T3				
Pre-existing cancer worries	0.32	0.00	0.10	0.66

The multiple regression analysis showed that worries about cancer at T2 were best predicted by pre-existing cancer worries, a high risk perception, and being single (explained variance was 31 % and AUC was 80 %). At T3, worries about cancer were best predicted again by pre-existing cancer worries and being single, but also by an unfavorable test result (explained variance was 23 % and AUC was 77 %). Emotional distress at T2 was best predicted by prior emotional distress (explained variance was 18 % and AUC was 72 %). At T3, emotional distress was best predicted by pre-existing cancer worries (explained variance was 10 % and AUC was 66 %). Thus, hypothesis 2 was partially supported.

## Discussion

As expected, after genetic testing for hereditary cancer, counselees with an unfavorable result show no more emotional distress than those with a favorable result. This was demonstrated shortly after the DNA test result was known, as well as 4–6 weeks later. However, counselees with an unfavorable result did ultimately have more concerns about cancer than those with a favorable result, only not directly after the test result was known, but 4–6 weeks later. These results suggest that an unfavorable result for hereditary cancer leads to a specific psychological impact in the form of more concerns about cancer a few weeks after the result is known. This may be explained by the fact that these counselees are confronted with a proven high lifetime risk of cancer. In prior research where *general* psychological distress was studied (e.g., Beran et al. 2008; Collins et al. 2007; Smith et al. 2008), these concerns about cancer could have been missed, which could lead to the erroneous conclusion that an unfavorable result carries no psychological distress. Although these studies have also used the Impact Event Scale (IES) as a specific cancer distress measure, this instrument could be incapable to measure worries about cancer, as it measures a stress reaction after a traumatic incident instead of worries about getting cancer in the future.

Worries about cancer after DNA testing were best predicted by four factors: pre-existing cancer worries, an unfavorable result, a high risk perception of getting cancer, and being single. Pre-existing emotional distress did not predict cancer worries. Strikingly, a high risk perception of getting cancer had an effect on worries about cancer only shortly after the result was made known, while an unfavorable result only had an effect 4–6 weeks later. The finding that single people are more worried about cancer than those with a partner was not predicted, because this factor was not known from earlier research. Single people may have more cancer worries because they have less social support. Worries about cancer were better predicted by pre-existing worries about cancer than by the actual genetic test result, which is an important consideration for counseling and corresponds with previous results (Meiser 2005; Van Oostrom et al. 2007). Our findings about the influence of an unfavorable result, high risk perception and pre-existing cancer worries on worries about getting cancer corresponds with prior research where a wide range of prognostic variables have been examined (Den Heijer et al. 2013; Gopie et al. 2012; Van Oostrom et al. 2007).

Although the explained variance was low, these prognostic variables may enable us to identify those counselees who are more likely to be affected by cancer worries immediately after the DNA test result is made known and some weeks later. In contrast, it is more difficult to predict who will develop emotional distress after genetic testing. Pre-existing emotional distress was the only predictive factor immediately after the result was known, whereas after 4–6 weeks, pre-existing worries about cancer appeared to be the only relevant prognostic factor. The DNA test result can play an indirect role in emotional distress, since an unfavorable result led to more worries about cancer and such worries also increase emotional distress.

### Study Limitations

The strengths of this study are its prospective design, the large series of measurements, the homogenous population, the large study sample, the broad range of prognostic variables considered, and the double outcome measure. One limitation is that the influence of social factors, personality and coping style were not examined. Another limitation may be that, although this study population contained the most prevalent hereditary cancer syndromes (*BRCA* and Lynch syndrome), it is not clear whether these findings can be generalized to other, less common, forms of hereditary cancer. The participants came from families in which the causative gene mutation had been identified. Thus, there were no counselees with an “unclear test result” or “variant type of result” in this study, which means that it is unknown how these findings apply in these cases. Some measure instruments had low reliabilities, which means that those results should be interpreted with caution. Finally, the follow-up measure was 4–6 weeks after the DNA result was known, which is relatively short. It is unclear whether the measured effects persist in the longer term. The studies available on long-term effects show that distress decreases with time (Foster et al. 2007; Halbert et al. 2011). The fact that counselees with an unfavorable outcome were, on average, younger than counselees with a favorable outcome, can be explained by the age-related penetrance of these diseases. This may also explain why these counselees had a higher prior risk perception. Furthermore, because the counselees with a favorable outcome were, on average, older, it is understandable that they more often had children.

### Practice Implications

In genetic counseling for individuals at 50 % risk of carrying an inherited *BRCA1/2* mutation or Lynch syndrome more focus should be placed on cancer worries besides general distress. Genetic counselors should offer additional guidance to counselees with many worries about cancer or emotional distress prior to genetic testing, to those with a high risk perception of getting cancer, and to those with little social support. If counselees would like to receive treatment against cancer worries, this could be offered in the form of cognitive interventions, where counselees learn to contest disturbing thoughts and focus more on reassuring thoughts. These kinds of interventions could be taught to genetic counselors by psychologists and could be offered to counselees who score high on cancer worries in screening instruments for genetic testing. Although no research is yet available about cognitive interventions to lessen cancer worries, cognitive methods are successful in the treatment of generalized anxiety disorder (Hanrahan et al. 2013), a disorder where disturbing worries are the main symptom.

### Research Recommendation

Knowing that worries about cancer play a major role before and after genetic testing, future research should assess the most effective ways of coping and the best guidance regarding these worries. Future research could also clarify what happens to patients with cancer worries in the longer term. As it appears that single people have more worries about cancer, it could be worthwhile to study the influence of partner status and social support in future research.

## Conclusion

The psychological impact of an unfavorable genetic test result is considerable if it measured as “worries about cancer” instead of “general distress.” Counselees with many cancer worries and emotional distress prior to genetic testing, with an unfavorable DNA test result, and those with a high risk perception of getting cancer or a weak social network appear to have a higher risk of psychological problems. The present study confirms and extends previous findings (Den Heijer et al. 2013; Gopie et al. 2012; Van Oostrom et al. 2007) and adds to the understanding of the variables that can be used to predict the development of psychological problems after genetic testing. Studying prognostic variables that can predict distress can help in the validation process of recently developed, risk factor screening instruments for genetic testing (Cella et al. 2002; Eijzenga et al. 2014; Esplen et al. 2013; Phelps et al. 2010; Read et al. 2005).
